# Real life use of vasopressin in patients with cardiogenic shock: a retrospective cohort analysis

**DOI:** 10.1186/s13054-023-04574-8

**Published:** 2023-07-19

**Authors:** Maxime Nguyen, Vivien Berthoud, Alexis Rizk, Bélaïd Bouhemad, Pierre-Grégoire Guinot

**Affiliations:** 1 Department of Anesthesiology and Intensive Care, Dijon University Hospital, 21000 Dijon, France; 2 University of Burgundy and Franche-Comté, LNC UMR1231, 21000 Dijon, France; 3 INSERM, LNC UMR1231, 21000 Dijon, France; 4 FCS Bourgogne-Franche Comté, LipSTIC LabEx, 21000 Dijon, France

 In the intensive care unit (ICU), vasopressin is administered as a second-line vasopressor. Although vasopressin has been extensively studied in patients with septic shock, there is a scarcity of data regarding its use in patients with cardiogenic shock. This is noteworthy because patients with cardiogenic shock often possess limited cardiac reserve, which can lead to challenges in augmenting cardiac workload when confronted with increased afterload. Consequently, understanding the hemodynamic response to vasopressin in this specific patient population may be important. Here, we studied the clinical response to vasopressin in patients suffering from cardiogenic shock and refractory vasoplegia.

We conducted a retrospective study in a cardiac ICU at Dijon University Hospital, France. Informed consent was obtained (IRB 00010254-2023-025). All consecutive patients admitted to our ICU from July 2020 to September 2022 were included if they were over 18 years old, diagnosed with cardiogenic shock, and treated with vasopressin. In our ICU, vasopressin treatment was recommended for patients requiring high-dose vasopressors, and vasopressin was administered continuously at a rate of 0.01 to 0.06 IU min^−1^. The norepinephrine equivalent (NEE) dose was calculated [1]. A pressure response was defined as a decrease in NEE with a mean arterial pressure (MAP) higher than 65 mmHg at 6 h [2].

We analyzed data from 100 patients (76 males) who were treated with vasopressin (Fig. 1). The median age was 64 years [58;72], the SAPSII 55.0 [46;71]. The Society of Cardiovascular Angiography Interventions (SCAI) score were as follows: C (18%), D (55%), and E (27%). Main causes of cardiogenic shock were ischemic (26%), non-ischemic (20%), and post-cardiotomy (32%). 56% of the patients were supported by veno-arterial ECMO, 44% dobutamine and 7% epinephrine. All patients received norepinephrine, with a median dose of 1.44 [1.02;2.40] µg kg^−1^ min^−1^. Arterial lactate levels were 6.70 [3.65;10.9] mmol l^−1^. The time between initiation of norepinephrine and vasopressin administration was 8 [4;20] h. The maximum dose of vasopressin was 0.03 [0.03;0.04] IU min^−1^. Vasopressin effectively increased MAP and decreased NEE in the overall population (Fig. 1A and B), with 55% (51/93) of patients demonstrating a pressure response. The pressure response was not associated with SCAI classification, ECMO use, pH or lactate at baseline. At day 30, 79% of patients had died, primarily due to multi-organ failure (56 patients, 73%). The pressure response did not show a significant difference between survivors and deceased patients (67% vs. 51%, *p* = 0.32). However, from the 4 h time point onwards, there was a significant increase in mean arterial pressure (MAP) among the 30-day survivors (Fig. 1C). NEE doses exhibited differences between survivors and deceased patients at the 8 h and 12 h time points (Fig. 1D). Ischemic complications were observed in 29 patients (29%): 20% had acute mesenteric ischemia, 12% had skin ischemia, 10% had digital ischemia, and 3% had hyponatremia. The occurrence of ischemic complications was not associated with the early pressure response to vasopressin or vasopressin doses but was associated with longer durations of vasopressin administration (30 [13;56] vs. 48 [28;108] h, *p* < 0.01).

**Fig. 1:**
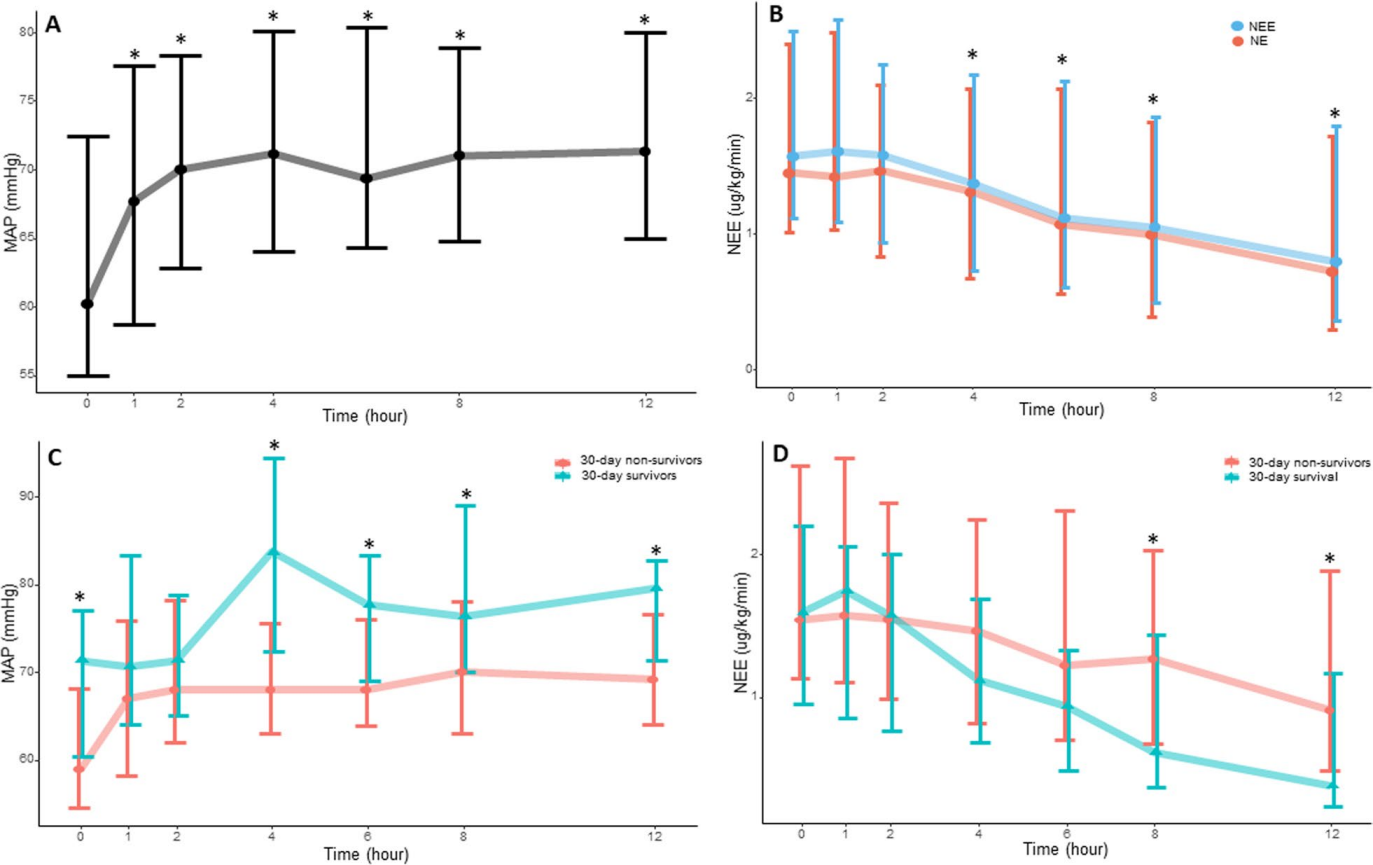
Pressure response to vasopressin in the overall population (**A** and **B**) and according to 30 day mortality (**C** and **D**). * in the A and B panel indicate significant differences from baseline. * in the C and D panel indicate between groups significant differences. * in panel D refers to Norepinephrine equivalent doses. *NEE* Norepinephrine equivalent; *MAP* Mean arterial pressure

In our cohort, 55% of patients were pressure responders to vasopressin, which is consistent with previous reports in septic shock [2]. However, this pressure response was not found to be associated with 30-day mortality in our cohort. Nevertheless, we observed that higher mean arterial pressure and lower NEE doses were linked to increased survival starting from the 4-h time point onwards. In addition to better perfusion resulting from higher arterial pressure, higher MAP may indicate greater cardiac reserve in patients with cardiogenic shock. Indeed, increased arterial load induced by vasopressin was likely to increase cardiac workload, and patients who couldn't adapt to this increased workload probably had decreased cardiac output (thus lower MAP). Furthermore, since vasopressin has no direct effect on cardiac contractility, the "isolated" vasoconstriction caused by vasopressin may further alter ventriculo-arterial coupling phenomenon in patients with lower cardiac reserve.

This study has several limitations. First, it is a retrospective study, and no causality can be inferred from our data. Second, the small number of survivors reduced the power of our analysis. Third, all of our patients received vasopressin, so the effect of vasopressin compared with a standard vasopressor could not be studied. Finally, the reporting of cardiac index and cardiac contractility monitoring would have provided a better understanding of the ventriculo-arterial coupling phenomenon, which appears to be a central issue in this population. However, we were unable to reliably extract this monitoring data from the records for too many patients.

## Data Availability

The datasets used and/or analysed during the current study are available from the corresponding author on reasonable request.

## Abbreviations

ICU: Intensive care unit
NEE: Norepinephrine equivalent
SCAI: Society of Cardiovascular Angiography Interventions
SAPS 2: Simplified acute physiology score
MAP: Mean arterial pressure
